# Different Susceptibility to the Parkinson's Toxin MPTP in Mice Lacking the Redox Master Regulator Nrf2 or Its Target Gene Heme Oxygenase-1

**DOI:** 10.1371/journal.pone.0011838

**Published:** 2010-07-28

**Authors:** Nadia G. Innamorato, Agnieszka Jazwa, Ana I. Rojo, Concepción García, Javier Fernández-Ruiz, Anna Grochot–Przeczek, Anna Stachurska, Alicja Jozkowicz, Jozef Dulak, Antonio Cuadrado

**Affiliations:** 1 Centro de Investigación Biomédica en Red sobre Enfermedades Neurodegenerativas (CIBERNED), Instituto de Investigación Sanitaria La Paz (IdiPaz), Madrid, Spain; 2 Departamento de Bioquímica e Instituto de Investigaciones Biomédicas “Alberto Sols” Consejo Superior de Investigaciones Científicas (CSIC), Universidad Autónoma de Madrid (UAM), Madrid, Spain; 3 Department of Medical Biotechnology, Faculty of Biochemistry, Biophysics and Biotechnology, Jagiellonian University, Krakow, Poland; 4 Departamento de Bioquímica y Biología Molecular, Instituto Universitario de Investigación en Neuroquímica, Facultad de Medicina, Universidad Complutense de Madrid (UCM), Madrid, Spain; The Mental Health Research Institute of Victoria, Australia

## Abstract

**Background:**

The transcription factor Nrf2 (NF-E2-related factor 2) and its target gene products, including heme oxygenase-1 (HO-1), elicit an antioxidant response that may have therapeutic value for Parkinson's disease (PD). However, HO-1 protein levels are increased in dopaminergic neurons of Parkinson's disease (PD) patients, suggesting its participation in free-iron deposition, oxidative stress and neurotoxicity. Before targeting Nrf2 for PD therapy it is imperative to determine if HO-1 is neurotoxic or neuroprotective in the basal ganglia.

**Methodology:**

We addressed this question by comparing neuronal damage and gliosis in Nrf2- or HO-1-knockout mice submitted to intraperitoneal injection of 1-methyl-4-phenyl-1,2,3,6-tetrahydropyridine (MPTP) for five consecutive days. Nrf2-knockout mice showed exacerbated gliosis and dopaminergic nigrostriatal degeneration, as determined by immunohistochemical staining of tyrosine hydroxylase in striatum (STR) and substantia nigra (SN) and by HPLC determination of striatal dopamine and 3,4- dihydroxyphenylacetic acid (DOPAC). On the other hand, the severity of gliosis and dopaminergic degeneration in HO-1-null mice was neither increased nor reduced. Regarding free-iron deposition, both Nrf2- and HO-1-deficient mice exhibited similar number of deposits as determined by Perl's staining, therefore indicating that these proteins do not contribute significantly to iron accumulation or clearance in MPTP-induced Parkinsonism.

**Conclusions:**

These results suggest that HO-1 does not protect or enhance the sensitivity to neuronal death in Parkinson's disease and that pharmacological or genetic intervention on Nrf2 may provide a neuroprotective benefit as add on therapy with current symptomatic protocols.

## Introduction

Despite our limited understanding about the etiopathology of Parkinson's disease it is accepted that oxidative stress is critically involved in the dopaminergic neuron death because the SN of PD patients exhibits increased levels of oxidized lipids, proteins and DNA and a decrease in the levels of glutathione (GSH) [1]. Sources of oxidant free radicals in the basal ganglia include alterations in complex I of the mitochondrial respiratory chain, oxidative metabolism of dopamine and reactive oxygen species released by microglia. MPTP, which is converted to the complex I inhibitor 1-methyl-4-phenylpyridine (MPP^+^) by MAO-B oxidation, induces Parkinsonism in humans, primates and mice [2]–[4]. Complex I inhibition results in incomplete oxygen reduction and in generation of potentially harmful ROS, including superoxide [5]–[8], hydrogen peroxide (H_2_O_2_) by action of superoxide dismutases and hydroxyl radicals generated by iron-mediated Fenton reaction [9]–[10]. Moreover, a neurotoxic role of NADPH oxidase from microglia has been demonstrated in MPTP treated mice [11]–[12]. Considering these strong evidences for a role of oxidant injury in PD, recently it has been speculated that reinforcement of redox homeostasis, which declines with age [13]–[14], could be of therapeutic use to provide a neuroprotective add on therapy together with well-established dopamine replacements.

The transcription factor Nrf2 has been pointed as an excellent candidate to prevent dopaminergic cell death and inflammation [15]. Nrf2 is the master regulator of genes containing Antioxidant Response Elements [16] in their promoters, which constitute the antioxidant and detoxifying phase II response. These genes include HO-1, NAD(P)H:quinone oxidoreductase-1 (NOQ1) and enzymes involved in GSH metabolism such as γ-glutamylcysteine synthetase, and glutathione S-transferase [17]–[19]. Nrf2 has a very short half life due to its interaction with the BTB-Kelch protein Keap1 that promotes Nrf2 degradation by the proteasome [20]. Physiological or pharmacological stimuli promote the dissociation of the Nrf2–Keap1 complex resulting in stabilization of Nrf2, translocation to the nucleus and induction of phase II cytoprotective genes [21]–[22]. Based on the large number of experimental evidences that support this model, dissociation of Nrf2 from Keap1 by electrophilic drugs provides a major strategy to increase Nrf2 transcriptional activity.

A crucial role of Nrf2 has been demonstrated in protection of dopaminergic neurons against oxidative stress, detoxification of mitochondrial complex I inhibitors and down-regulation of genes involved in the brain innate immune response. We and others have demonstrated that Nrf2-null (Nrf2^−/−^) mice are more sensitive to MPTP treatment [23]–[25]. Moreover, Nrf2^−/−^ mice exhibit exacerbated inflammation compared to wild type littermates as determined by release of pro-inflammatory cytokines in basal ganglia [25]. However, while Nrf2 holds promise for PD therapy, a very important concern is to determine the role of one of its target genes, HO-1 in PD pathology.

The inducible phase II enzyme HO-1, together with the constitutively expressed isoenzyme HO-2, degrades free heme into three products: carbon monoxide (CO), biliverdin (which is rapidly converted to bilirubin), and free iron [26]–[28]. At low concentrations at least CO and bilirubin exert cytoprotective and anti-inflammatory activities [29]. In fact, up-regulation of HO-1 expression confers adaptive survival response to oxidative and inflammatory insults both *in vitro* and *in vivo* [24] [30]–[33]. By contrast, the relevance of iron in PD etiopathology is well documented in patients and in animal models. For instance, iron-deficient rats are resistant to 6-OHDA neurotoxicity [34]. Moreover, iron chelators attenuate 6-OHDA- [35] and MPTP-induced [36] toxicity. It has been suggested that dopaminergic degeneration is a result of excessive redox-active metals within the SN, which initiates a cascade of events, such as α-synuclein oligomerization, mitochondrial dysfunction, cytotoxicity, and a rise of cytosolic free calcium [16]. In postmortem biopsies, immunohistochemical analysis indicates a moderate increase in HO-1 protein levels in the cytoplasm of dopaminergic neurons of SN of PD patients, compared to control individuals. Moreover, there is a potent HO-1 immunoreactivity in Lewy bodies [9] [37]. These evidences have led to the speculation that increased HO-1 levels might be the main responsible for the accumulation of free iron and subsequent damage to the nigrostriatal system [38].

Therefore, in order to determine the therapeutic value of Nrf2-activating drugs for PD it is necessary to determine if HO-1, which will be up-regulated by Nrf2 activators through Nrf2-depedent transcription, has a neuroprotective or neurotoxic role. We have addressed this question in HO-1-knockout (HO-1^−/−^) mice and Nrf2^−/−^ mice submitted to the sub-acute model of MPTP. Our results, present two new findings: 1) HO-1^−/−^ mice are similarly sensitive to MPTP as wild type littermates, suggesting that the increase in free iron, at least in MPTP-induced parkinsonism is not related to HO-1. 2) Nrf2 is more efficient than HO-1 in eliciting a neuroprotective and anti-inflammatory response to MPTP indicating that, in addition to HO-1, other phase II enzymes are also required for an effective neuroprotection.

## Materials and Methods

### Animals and treatments

All animal protocols were approved by the Ethical Committee for Research of the Universidad Autónoma de Madrid and by the Institutional Animal Care and Use Committee at the Jagiellonian University following institutional, Spanish, Polish and European guidelines (Boletín Oficial del Estado (BOE) of 18 March 1988; and 86/609/EEC, 2003/65/EC European Council Directives). Animals were housed at room temperature under a 12 h light-dark cycle. Food and water was provided *ad libitum*. Mouse genotyping was done according to [39] and [40]. Six months-old male wild type C57BL/6 mice and Nrf2-knockout littermates (courtesy of Dr Masayuki Yamamoto, Sendai, Japan) and C57BL/6×FVB mice and HO-1-knockout littermates (courtesy of Dr. Anupam Agarwal, Birmingham, USA) were used. MPTP (Sigma-Aldrich; 30 mg/kg) was prepared in saline solution just before use. The compound was administered by intraperitoneal (i.p.) injection once a day for 5 consecutive days. Once the experimental schedule was completed, animals were anesthetized with 8 mg/kg ketamine and 1.2 mg/kg xylazine and perfused.

### Analysis of mRNA levels by semiquantitative PCR

Total RNA was extracted using TRIzol reagent according to the manufacturer's instructions (Invitrogen). One µg of RNA from the different treatments was reverse-transcribed in 20 µl using High Capacity RNA-to-cDNA Kit (Applied Biosystems) according to manufacturer's instructions. Semiquantitative amplification of cDNA was performed in 40 µl of Go *Taq* Flexi Colorless PCR buffer (Promega) containing 0.5 U of Go *Taq* Flexi DNA polymerase (Promega) and 30 pmol of synthetic gene-specific primers. Primer sequences were: HO-1 forward 5′-CACAGATGGCGTCACTTCCGTC-3′; HO-1 reverse, 5′-GTGAGGACCCACTGGGAGGAG-3′; β-actin forward, 5′-TCCTTCCTGGGCATGGAG-3′; β-actin reverse, 5′-AGGAGGAGCAATGATCTTGATCTT-3′. To ensure that equal amounts of cDNA were added to the PCR, the β-actin housekeeping gene was amplified. PCR cycles (optimized for every gene) proceeded as follows: initial denaturation for 4 min at 94°C, 1 min at 94°C (denaturation), 1 min at 58°C (annealing), and 1 min at 72°C (elongation). The amplified PCR products were resolved in 5% PAGE and visualized with SYBR Safe DNA Stain (Invitrogen).

### Striatal DA and DOPAC

Striata were homogenized in 50 volumes of ice-cold 0.2 N perchloric acid containing 0.2 mM sodium disulphide and 0.45 mM EDTA. Dihydroxybenzylamine was added as an internal standard. The homogenates were then centrifuged and the supernatants injected into the HPLC system. This consisted of a Spectra-Physics 8810 isocratic pump. The column was an RP-18 (Spherisorb ODS-2; 125 mm, 4.6 mm, 5 µm particle size; Waters, Massachusetts, USA). The mobile phase consisted of 100 mm citric acid, 100 mm sodium acetate, 1.2 mm heptane sulphonate, 1 mm EDTA and 7% methanol (pH 3.9) and the flow rate was 0.8 ml/min. The effluent was monitored with a coulochemical detector (Coulochem II, ESA) using a procedure of oxidation and reduction (conditioning cell, +360 mV; analytical cell no. 1, +50 mV; analytical cell no. 2, 340 mV). The signal was recorded from the analytical cell no. 2, with a sensitivity of 50 nA (10 pg per sample), on a Spectra-Physics 4290 integrator, and DA and DOPAC levels were obtained as area under the peaks, corrected for the amount of total protein in the tissue sample.

### Immunohistochemistry

Animals were perfused through the left ventricle with saline solution, followed by 4% paraformaldehyde in 0.1 M phosphate buffer, pH 7.4, for 15 min. Brains were removed and cryoprotected by soaking in 30% sucrose solution in phosphate buffer until they sank. Parallel series of 40-µm-thick coronal sections were obtained in a freezing microtome. Sections from control and experimental animals were processed with the same solutions and processing times. Sections were rinsed in 100 mM Tris–HCl, pH 7.6, and 225 mM NaCl (TBS). Antigen retrieval was performed by microwave heating of the sections for 15–20 min in 10 mM sodium citrate buffer, pH 6.0. Tissue peroxidase was inactivated by incubating in 10% methanol and 3% hydrogen peroxide in TBS for 30 min. After three washes in TBS sections were incubated for 3 h in blocking solution (10% goat serum, 0.3% Triton X-100 in TBS), and then for 48 h at 4°C with mouse anti-tyrosine hydroxylase (TH), rabbit anti-glial fibrillar acidic protein (GFAP) (1∶250; Millipore, Billerica, MA) or anti-Iba1 (1∶1000; Abcam). Sections were rinsed in TBS, then incubated with rabbit anti-mouse or anti-rabbit secondary antibodies at 1/5000 dilution for 1 h at room temperature (Vector Labs). Immunoreagents were diluted in 1% goat serum and 0.2% Triton X-100 in TBS. Sections were subsequently developed by avidin-biotin peroxidase complex system following manufacturer's instructions (ABC Kit, Vector Labs). Sections were mounted on gelatin-coated slides, air-dried and finally dehydrated in graded alcohols, cleared in xylene and coverslipped. Control sections were treated with the same protocol but omitting the primary antibody.

### Stereological quantification

Cell counts were performed every four sections (40 µm thick) through the SN using Stereo Investigator Software (MicroBrightfield, Colchester, VT, USA) attached to an E800 Nikon microscope (Nikon, Montreal, QC, Canada). SN, excluding ventral tegmental area (VTA), was delineated in low magnification (5X objective) and a point grid was overlaid onto each section. For Nissl staining, TH-stained sections were rehydrated in xylene and graded alcohols and incubated in Nissl's solution (0.1% Cresyl Violet (Sigma), 2.5 ml acetic acid 10%) at room temperature for 15 min. Sections were rinsed in deionised water for 5 min and dehydrated in graded alcohols, cleared in xylene and coverslipped. Stained cells were counted by the optical fractionator method at high magnification (63X objective, N.A. 1.25) distinguishing between TH-positive (TH and Nissl positive) and TH-negative (Nissl positive). TH- and Nissl-stained neurons were counted only when their nuclei were optimally visualized within one focal plane. Nissl-stained neurons were differentiated from non-neuronal cells by clearly defined nucleus, cytoplasm, and a prominent nucleolus. Total numbers of neurons in the SN were calculated as described in [41].

### DAB-enhanced Perls iron staining

Sections were rinsed in deionized water for 30 min and incubated in Perls solution (5% potassium ferrocyanide : 5% HCl, 1∶1, V∶V) at room temperature for 30 min. The reaction was stopped by rinsing in deionized water for 30 min. Staining was enhanced by incubation in 0.5% 3′-3′-diaminobenzidine tetrahydrochloride (Sigma-Aldrich) in Tris-ClH buffer, pH 8.0, for 20 min, and then developed in the same buffer containing 0.003% hydrogen peroxide (Sigma-Aldrich) for 20 min. Finally, sections were incubated in Nissl's solution (0.1% Cresyl Violet (Sigma), 2.5 ml acetic acid 10%) at room temperature for 15 min, rinsed in deionized water for 5 min, dehydrated in graded alcohols, cleared in xylene and coverslipped.

### Immunofluorescence

Brains were fixed as described for immunohistochemistry. Parallel series of 40-µm-thick coronal sections were obtained in a freezing microtome. Sections were rinsed in TBS. After three washes, the sections were incubated for 3 h in blocking solution (10% goat serum, 0.3% Triton X-100 in TBS), and then for 48 h at 4°C in the following primary antibodies: rabbit anti-TH (1∶500; Millipore) and mouse anti-HO-1 (1∶100, Stressgen). Sections were rinsed in TBS and washed three times and then incubated with secondary antibodies for 45 min: Alexa Fluor 488 anti-rabbit or Alexa Fluor 546 anti-mouse at a 1∶100 dilution (Invitrogen). Immunoreagents were diluted in 1% goat or rabbit serum and 0.2% Triton X-100 in TBS. Sections were mounted on gelatin-coated slides, air-dried and finally dehydrated in graded alcohols, cleared in xylene and coverslipped. Control sections were treated with the same protocol but omitting the primary antibody. The fluorescence images were captured using appropriate filters in a Leica DMIRE2TCS SP2 confocal microscope (Nussloch, Germany). The lasers used were Ar 488 nm for green fluorescence and Ar/HeNe 543 nm for red fluorescence.

### Immunoblotting

STR and VMB were removed rapidly and homogenized on ice with lysis buffer (Tris-HCl, pH 7.5, 20 mM; NaCl, 137 mM; NaF, 20 mM; sodium pyrophosphate, 1 mM; Na_3_VO_4_, 1 mM; Nonidet P-40,1%; glycerol, 10%; phenyl methyl sulphonyl fluoride, 1 mM; and leupeptin, 1 µg/ml). Protein extracts were cleared by centrifugation and 30 µg protein were resolved by SDS-PAGE and transferred to Immobilon-P membranes (Millipore). Blots were analyzed with the appropriate antibodies: anti-TH (1∶2000; Millipore), anti-GFAP (1∶2000; Dako, Denmark), anti-Iba1 (1∶1000; Abcam, Cambridge, UK), anti-HO-1 (1∶2000; Millipore), anti-NQO1 (1∶500, Abcam), anti-HO-2 (1∶500, Stressgen), anti-GCL-C, anti-GCL-M (both were a kind gift of Dr Terrance Kavanagh, University of Washington, USA) and anti-β-actin (1∶1000; Santa Cruz Biotechnology). Appropriate peroxidase-conjugated secondary antibodies (1∶10,000) were used to detect the proteins of interest by enhanced chemiluminescence (Advanced ECL, GE Healthcare).

### Quantification and statistics

Different band intensities corresponding to immunoblot detection of protein samples were quantified using the MCID software (MCID, Cambridge, UK). Two-way ANOVA with Bonferroni's *post hoc* test or Student's *t* test was used to assess differences between groups.

## Results

### MPTP induces HO-1 expression in mouse substantia nigra

**Figure 1:**
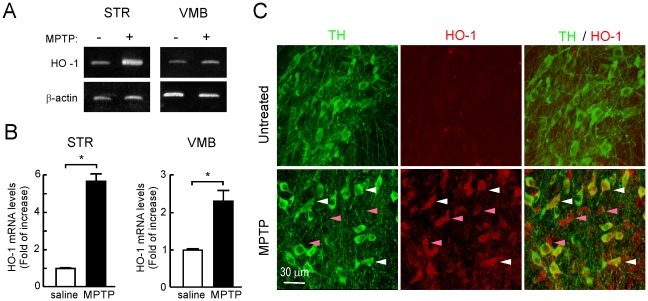
MPTP induces mRNA and protein HO-1 levels in STR and VMB. *A,* semiquantitative PCR in STR and VMB after MPTP treatment. *Upper panel:* HO-1 mRNA levels. *Lower panel:* β-actin mRNA levels. *B,* densitometric quantifications (arbitrary densitometry units) from representative PCRs of *A* after normalization by β-actin densitometry units.Results are representative of 5–10 animals per group. Values represent mean ± SD. A Student's *t* test was applied to determine the significance of biochemical differences among groups. Asterisks denote significant differences between treatments with *P*<0.05. *C,* double immunofluorescence of 40 µm-thick coronal sections from VMB of saline- and MPTP-treated mice, stained anti-TH (green) and HO-1 (red) antibodies. White arrows point some cells which are positive for both TH and HO-1expression. Pink arrows show nerve cells positive for HO-1 but not for TH.

Mice were submitted to the sub-acute model of MPTP administration consisting on one daily intraperitoneal injection of MPTP (30 mg/kg) for five consecutive days. HO-1 transcript levels in stratum (STR) and ventral midbrain (VMB) were analyzed 16 h after the last MPTP injection by semiquantitative PCR. As shown in Fig. 1A and 1B, messenger HO-1 levels were increased at both locations after the MPTP treatment. We also analyzed HO-1 expression by immunofluorescence in SN by double staining with anti-TH and anti-HO-1 antibodies (Fig. 1C). In control saline-treated mice, HO-1 immunoreactivity was barely detectable, in agreement with the concept that this inducible enzyme exhibits very low basal level of expression. By contrast, in MPTP-treated mice, HO-1 was increased in several nerve cells including some TH-positive dopaminergic neurons. Therefore, the MPTP mouse model reproduces the increase in HO-1 expression that has been reported in the human PD pathology.

### MPTP induction of nigrostriatal dopaminergic degeneration on HO-1^−/−^ and Nrf2^−/−^ mice and their wild type littermates

**Figure 2:**
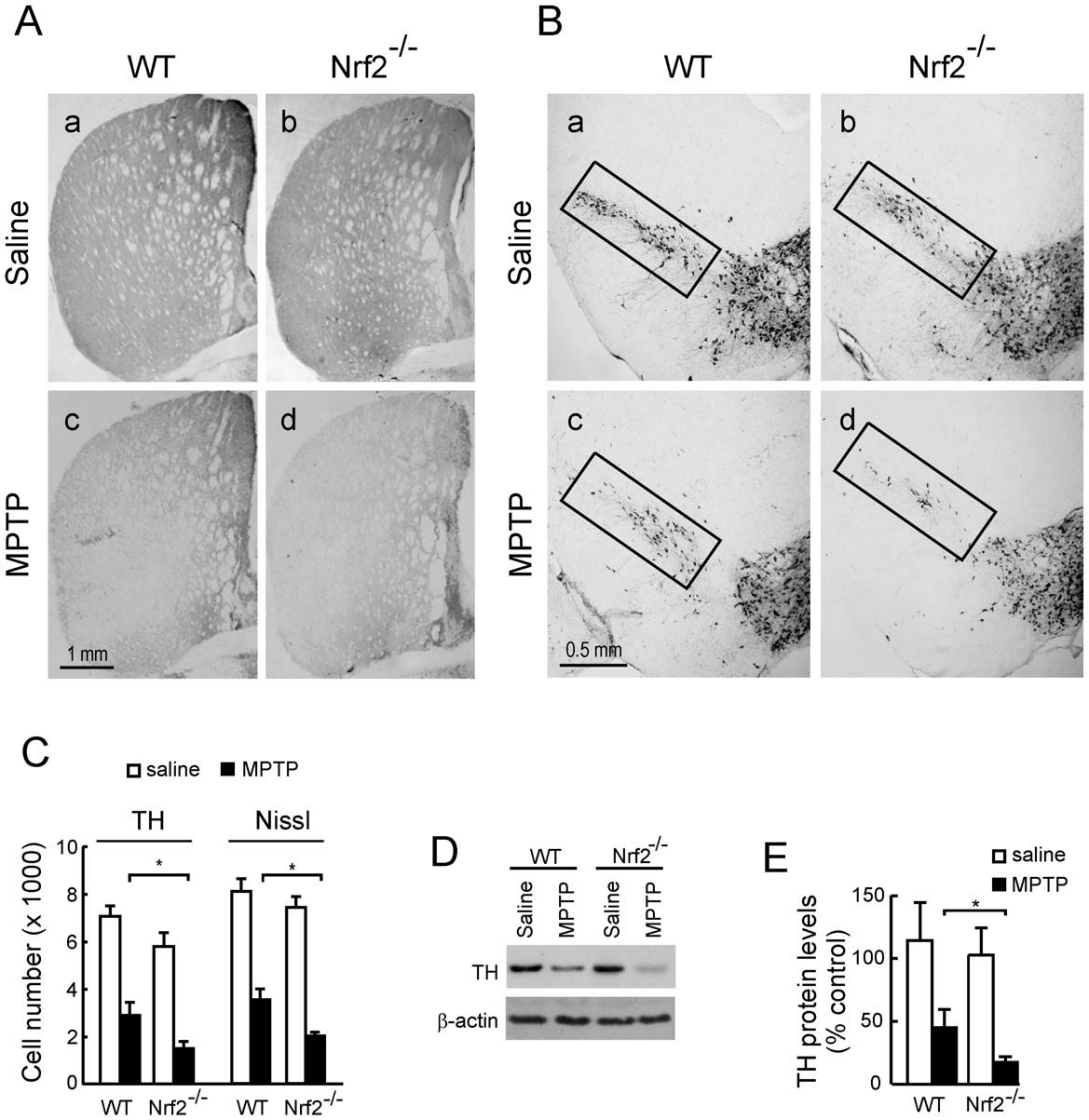
Sub-acute MPTP treatment induces a more profound lesion in STR and SN of Nrf2^−/−^ mice in comparison to their wild type littermates. Animals received one daily i.p. injection of saline or MPTP (30 mg/kg) for five consecutive days. Pictures show representative coronal sections, 40-µm thick, from VMB and STR stained with anti-TH antibody and counterstained with Nissl. *A*, Dopaminergic denervation in STR. *B*, Loss of dopaminergic neurons in VMB. Rectangles indicate SN. *C*, Stereological quantification of TH-immunoreactive neurons and Nissl positive neurons in SN. *D*, immunoblot of STR protein lysates. *Upper panel*, anti-TH antibody. *Lower panel*, anti-β-actin antibody showing similar protein load per lane. *E*, densitometric quantifications (arbitrary densitometry units) from representative immunoblots of D after normalization by β-actin densitometry units obtained from the same immunoblot. Results are representative of 5–10 animals per group. Values represent mean ± SD. Two-way ANOVA followed by Bonferroni's test was applied to determine the significance of biochemical differences among groups. Asterisks denote significant differences between treatments with *P*<0.05.

**Figure 3:**
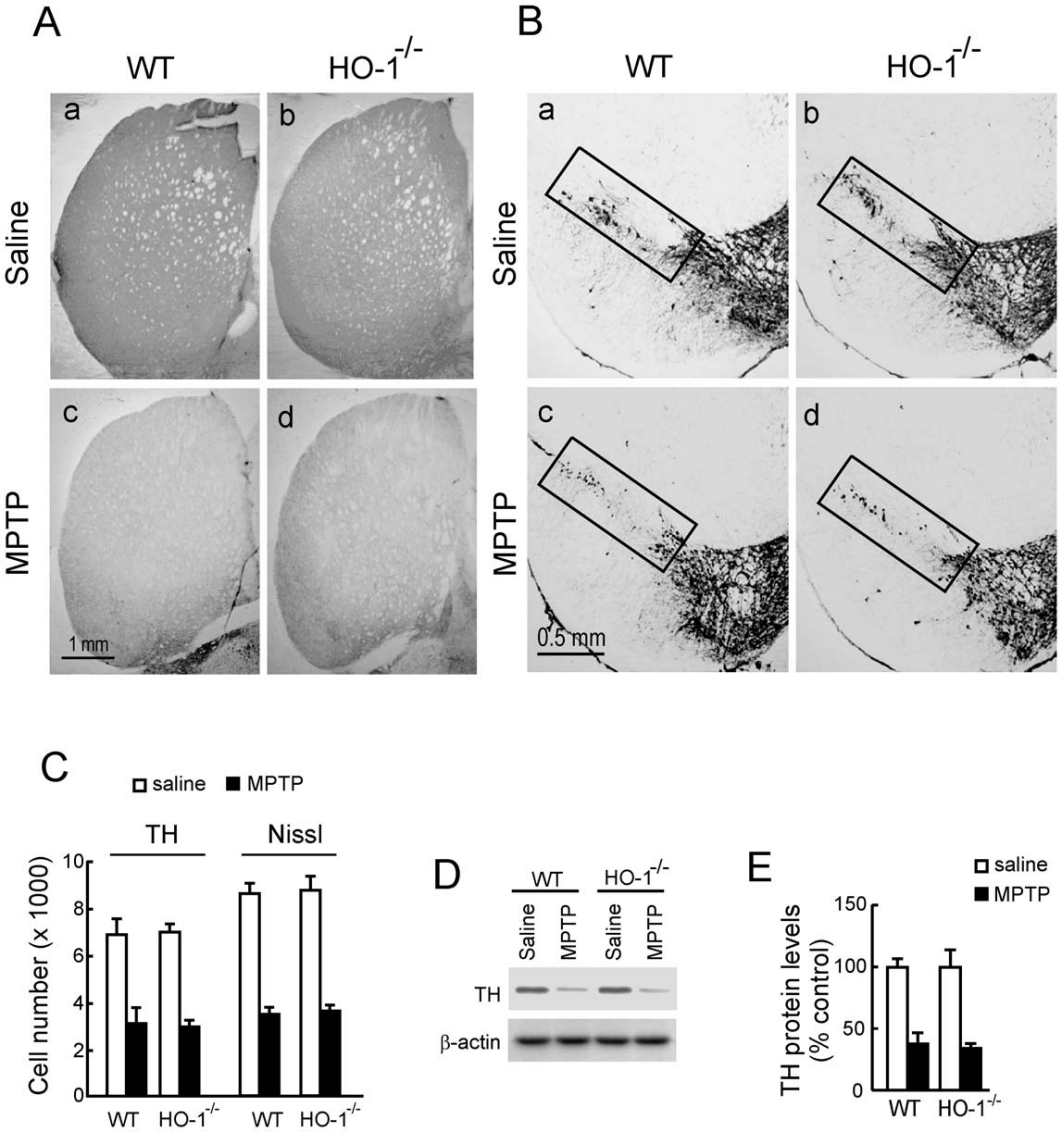
MPTP treatment induces a similar lesion in striatum-substantia nigra of HO-1^−/−^ mice and their wild type littermates. Animals received one daily i.p. injection of saline or MPTP (30 mg/kg) for five consecutive days. Pictures show representative coronal sections, 40-µm thick, from VMB and STR stained with anti-TH antibody and counterstained with Nissl. *A*, Dopaminergic denervation in STR. *B*, Loss of dopaminergic neurons in VMB. Rectangles indicate SN. *C*, Stereological quantification of TH-immunoreactive neurons and Nissl positive neurons in SN. *D*, immunoblot of STR protein lysates. *Upper panel*, anti-TH antibody. *Lower panel*, anti-β-actin antibody showing similar protein load per lane. *E*, densitometric quantifications (arbitrary densitometry units) from representative immunoblots of D after normalization by β-actin densitometry units obtained from the same immunoblot.Results are representative of 5–10 animals per group. Values represent mean ± SD. Two-way ANOVA followed by Bonferroni's test was applied to determine the significance of biochemical differences among groups.

We analyzed TH immunoreactivity as a phenotypic marker for dopaminergic neuron bodies and fibers. Mice treated with 30 mg/kg/day of MPTP for 5 consecutive days exhibited a drastic reduction in TH-immunoreactivity and Nissl staining in both STR (Fig. 2A) and VMB (Fig. 2B), indicating damage onto the nigrostriatal tract. However, Nrf2*^−/−^* mice showed a more pronounced reduction in TH-immunoreactivity in comparison with their wild type littermates. Stereological counts of nigral dopaminergic neurons, scored from either TH-immunoreactive and Nissl-stained sections, indicated that the Nrf2^−/−^ mice used in this study (6-months old) have about 15% fewer nigral dopaminergic neurons than their Nrf2^+/+^ littermates. Interestingly, in 2 weeks-old animals there was not any significant difference in cell counts or TH-staining (data not shown), therefore suggesting that Nrf2-defficiency leads to spontaneous dopaminergic degeneration. After the MPTP treatment the absolute loss in dopaminergic cells was greater in Nrf2^−/−^ mice compared to MPTP-treated wild type mice (Fig. 2C). However, changes in cell numbers between Nrf2^+/+^ and Nrf2 ^−/−^ mice were more similar when cell numbers were analyzed relative to their baseline controls. Thus the percentage of reduction between saline and MPTP-treated groups was 41% ± 5% in Nrf2^+/+^ mice v.s. 28% ± 3% in Nrf2^−/−^ mice (p<0.0046) for TH-stained cells and 42% ± 3% in Nrf2^+/+^ v.s. 30% + 2% in Nrf2^−/−^ mice (p<0.0009). Because the changes in nigrostriatal dopaminergic cell numbers were statistically significant but still modest when considered relative to their saline controls, these results were further quantified by densitometry of TH-stained STR sections (data not shown) and by immunoblot of STR protein lysates analyzed with anti-TH antibody (Fig. 2D and 2E) further supporting the concept that Nrf2*^−/−^* are more sensitive to MPTP. By contrast, HO-1*^−/−^* mice exhibited similar damage to the nigrostriatal pathway as their wild type littermates, as determined by TH-immunohistochemistry of STR (Fig. 3A) and VMB (Fig. 3B) sections, stereological counts of TH-immuoreactivity and Nissl staining (Fig. 3C), and by immunoblot quantification with anti-TH antibody (Fig. 3D and 3E). Therefore, while Nrf2*^−/−^* mice are more sensitive to MPTP-induced dopaminergic damage, HO-1*^−/−^* mice are similarly sensitive to this toxin as wild type mice.

### Striatal dopamine levels in Nrf2^−/−^, HO-1^−/−^ and wild type mice submitted to MPTP

**Figure 4:**
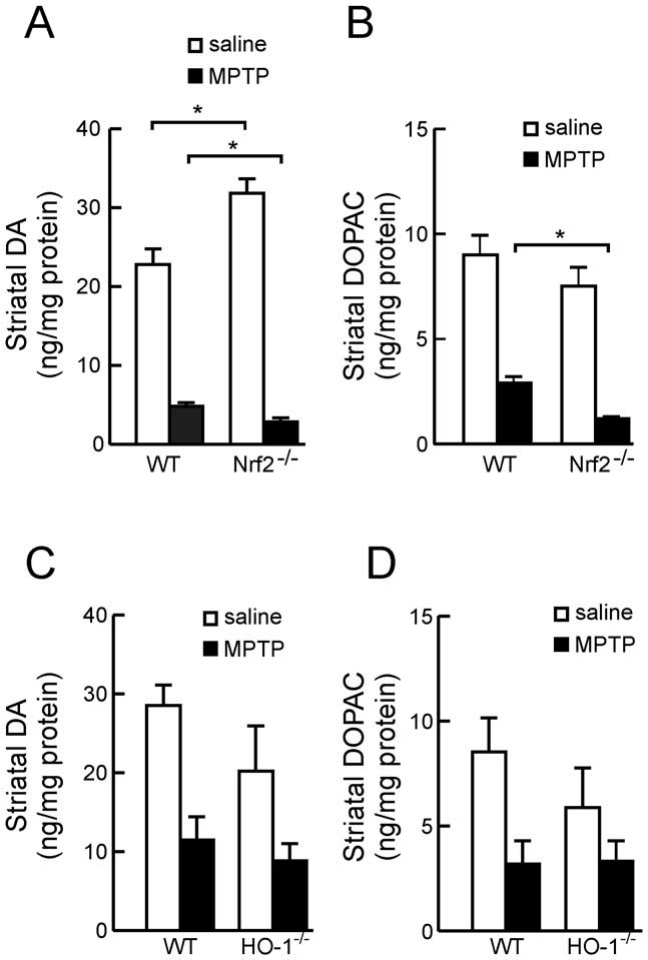
DA and DOPAC levels in STR of MPTP-treated Nrf2^−/−^ and HO-1^−/−^ and wild type littermate mice. Animals received one daily i.p. injection of saline or MPTP (30 mg/kg) for five consecutive days and striatal DA and DOPAC levels were determined by HPLC. *A and B,* the reduction in DA and DOPAC is more pronounced in MPTP-treated Nrf2^−/−^ mice than in MPTP-treated wild type littermates. *C and D,* the reduction in DA and DOPAC is similar between MPTP-treated HO-1^−/−^ mice and their wild type littermates. Values represent the mean ± SD from five samples. Two-way ANOVA followed by Bonferroni's test was applied to determine the significance of biochemical differences among groups. Asterisks denote significant differences between treatments with *P*<0.05.

Next, we analyzed striatal levels of DA and its major intra-neuronal degradation product, 3,4-dihydroxyphenylacetic acid (DOPAC) by HPLC. The basal striatal level of DA was slightly increased in Nrf2*^−/−^* mice compared to their wild type controls. We attribute this small increase to a compensatory mechanism for loss of striatal dopaminergic fibers. The molecular mechanism underlying this compensatory effect might be related to a decrease in the metabolic degradation of this neurotransmitter in the Nrf2-null mice. Thus, while DA levels go up, DOPAC levels seem to be more stable. The ratio DOPAC/DA, which is indicative of dopamine turnover in dopaminergic terminals is about 0.4±0.1 sd for wild type mice and lower (0.23±0.06 sd) for knockout mice, therefore suggesting that dopamine is somewhat more stable in knockout mice. Administration of 30 mg/kg/day of MPTP decreased the content of dopamine and DOPAC in all groups of animals included in the study (Figure 4). However, the DA and DOPAC depletion was more pronounced in Nrf2*^−/−^* than in HO-1*^−/−^* mice compared to their wild type control animals exposed to MPTP. These results further confirm that striatal dopaminergic transmission is more impaired in Nrf2*^−/−^* mice than in HO-1*^−/−^* mice.

### Gliosis in Nrf2^−/−^, HO-1^−/−^ and wild type mice submitted to MPTP

**Figure 5:**
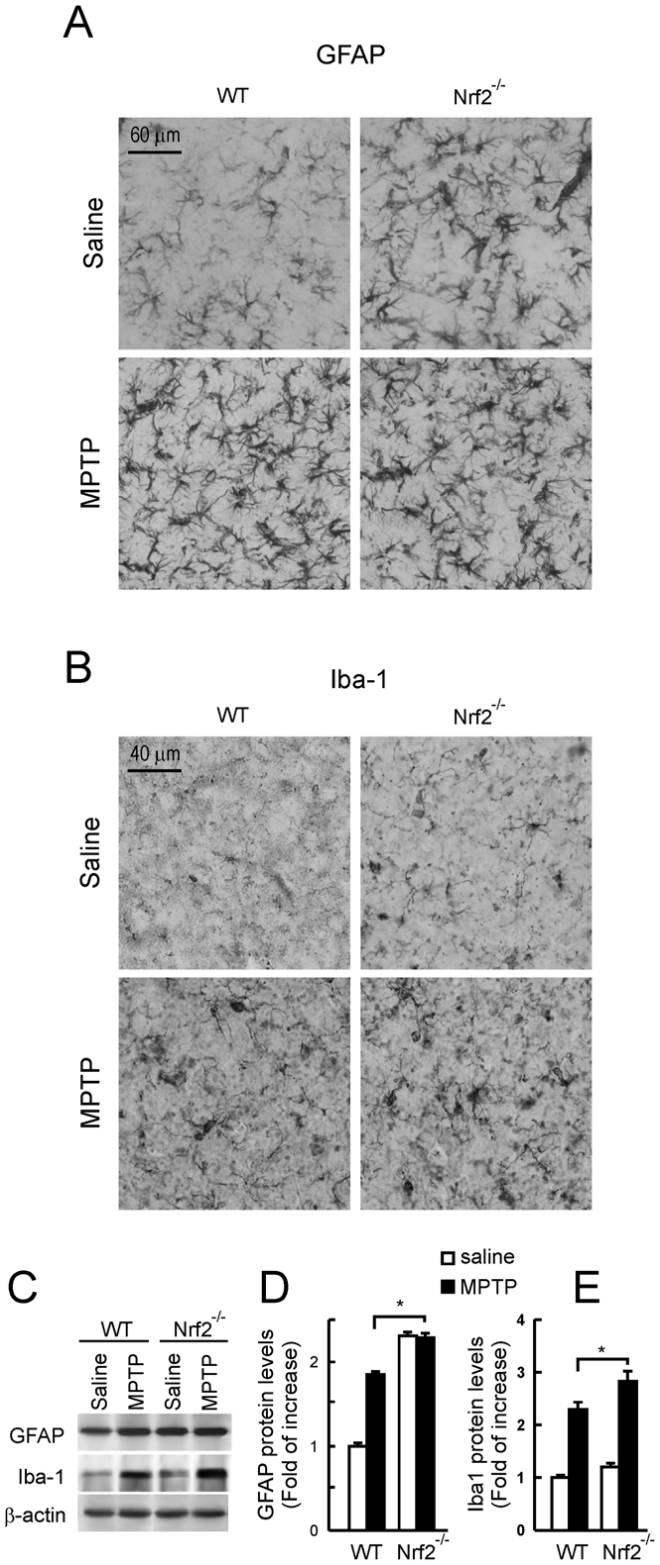
Nrf2^−/−^ mice show increased microglial activation compared to wild type littermates in response to sub-acute MPTP treatment. Immunohistochemistry was done in 40-µm thick coronal section of STR with anti-GFAP (astroglia) and anti-Iba1 (microglia) antibodies. *A,* immunohistochemical staining of STR with anti-GFAP antibody showing increased basal and MPTP-induced astroglia in Nrf2^−/−^ mice compared to wild type littermates. *B,* immunohistochemical staining of STR with anti-Iba1 antibody showing increased microglial activation in MPTP-treated Nrf2^−/−^ mice compared to wild type littermates. *C,* immunoblots from STR. *Upper panels*, anti-GFAP antibody; *middle panels*, anti-Iba-1 antibody; *lower panels*, anti-β-actin antibody. *D and E*, densitometric quantifications (arbitrary densitometry units) from representative immunoblots of C after normalization by β-actin densitometry units obtained from the same immunoblot.Results are representative of 5–10 animals per group. Values represent mean ± SD. Two-way ANOVA followed by Bonferroni's test was applied to determine the significance of biochemical differences among groups. Asterisks denote significant differences between treatments with *P*<0.05.

First, 40 µm-thick coronal sections from STR of Nrf2^−/−^ and HO-1^−/−^ mice and their wild type controls submitted to saline of MPTP treatments, were stained with anti-GFAP or anti-Iba1 antibodies to analyze astroglia or microglia respectively. Then, immunoblot analysis of STR protein lysates was used for quantification of astrocyte (anti-GFAP) and microglial (anti-Iba-1) levels. As shown in Fig. 5A and 5B, Nrf2^−/−^ exhibited higher basal levels of both glial cell types compared to their wild type controls. Indeed, this basal increase could be also quantified by immunoblots (Fig. 5C). In the MPTP-treated Nrf2^−/−^ animals astrogliosis was higher than in their wild type controls but similar to their baseline, suggesting that astrocyte accumulation in their STR is close to saturation. Regarding microglia, we detected an increase in MPTP-treated Nrf2^−/−^ mice, both over baseline and over the MPTP-treated controls. Therefore, while these results are not conclusive for astrogliosis, they suggest that Nrf2^−/−^ mice are prone to MPTP-induced microgliosis.

**Figure 6:**
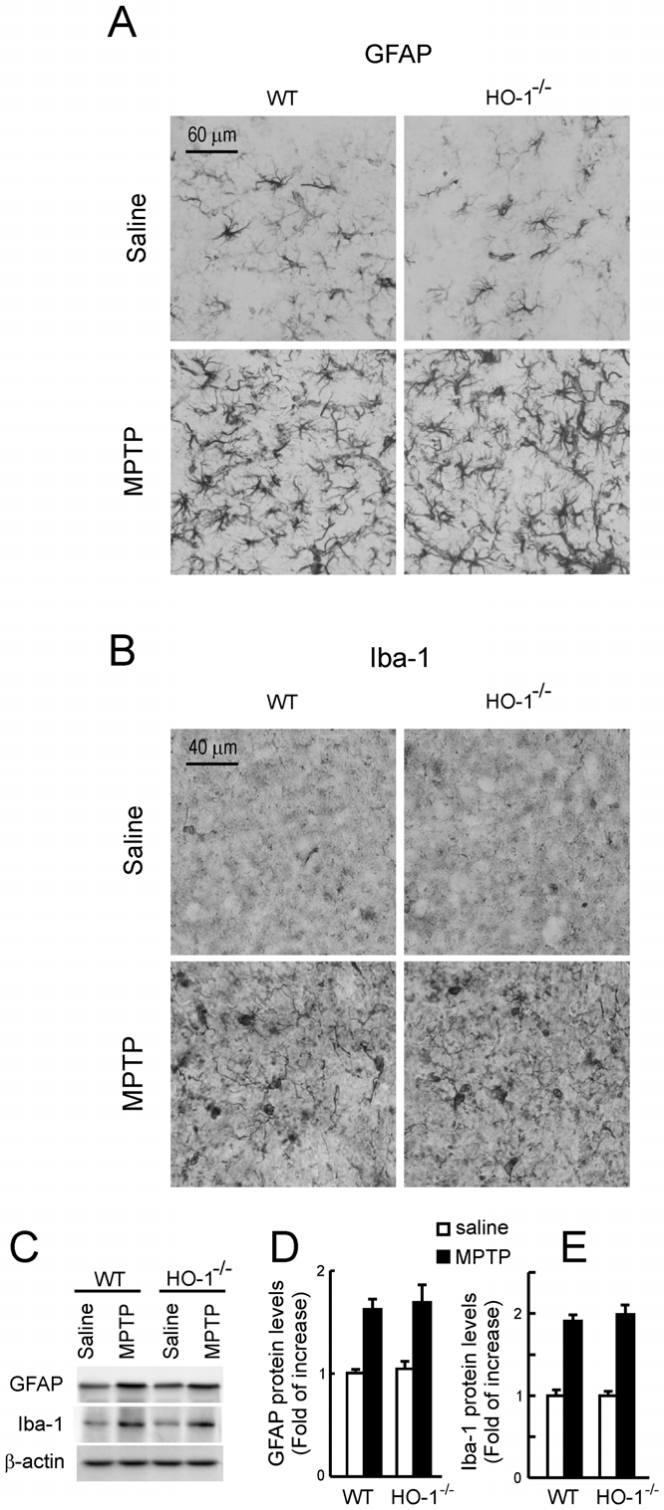
HO-1^−/−^ mice exhibit similar glial activation compared to wild type littermates in response to sub-acute MPTP treatment. Immunohistochemistry was done in 40-µm thick coronal section of STR with anti-GFAP (astroglia) and anti-Iba1 (microglia) antibodies. *A,* immunohistochemical staining of STR with anti-GFAP antibody showing no differences in astroglia in HO-1^−/−^ mice compared to wild type littermates in response to MPTP. *B,* immunohistochemical staining of STR with anti-Iba1 antibody showing no differences microglial activation in MPTP-treated HO-1^−/−^ mice compared to wild type littermates. *C,* immunoblots from STR. *Upper panels*, anti-GFAP antibody; *middle panels*, anti-Iba-1 antibody; *lower panels*, anti-β-actin antibody. *D and E*, densitometric quantifications (arbitrary densitometry units) from representative immunoblots of C after normalization by β-actin densitometry units obtained from the same immunoblots.Results are representative of 5–10 animals per group. Values represent mean ± SD. Two-way ANOVA followed by Bonferroni's test was applied to determine the significance of biochemical differences among groups.

Regarding HO-1^−/−^ mice, immunohistochemical analysis demonstrated a small number of astrocytes and microglia in the STR of saline-inoculated HO-1^−/−^ mice that was similar to their wild type controls (Figs. 6A and 6B). MPTP induced an increase in the number of both glial types in HO-1^−/−^ and wild type littermates. Immunoblot analysis indicated a close to 2 fold increase in striatal GFAP and Iba-1 levels in response to MPTP but there was no significant difference between in HO-1^−/−^ and wild type controls (Fig. 6C). These results suggest that Nrf2^−/−^ mice exhibit exacerbated basal and MPTP-induced gliosis and that HO-1*^−/−^* mice show a pattern of gliosis similar to their wild type littermates.

### Phase II antioxidant enzyme levels in Nrf2^−/−^, HO-1^−/−^ and wild type mice submitted to MPTP

**Figure 7:**
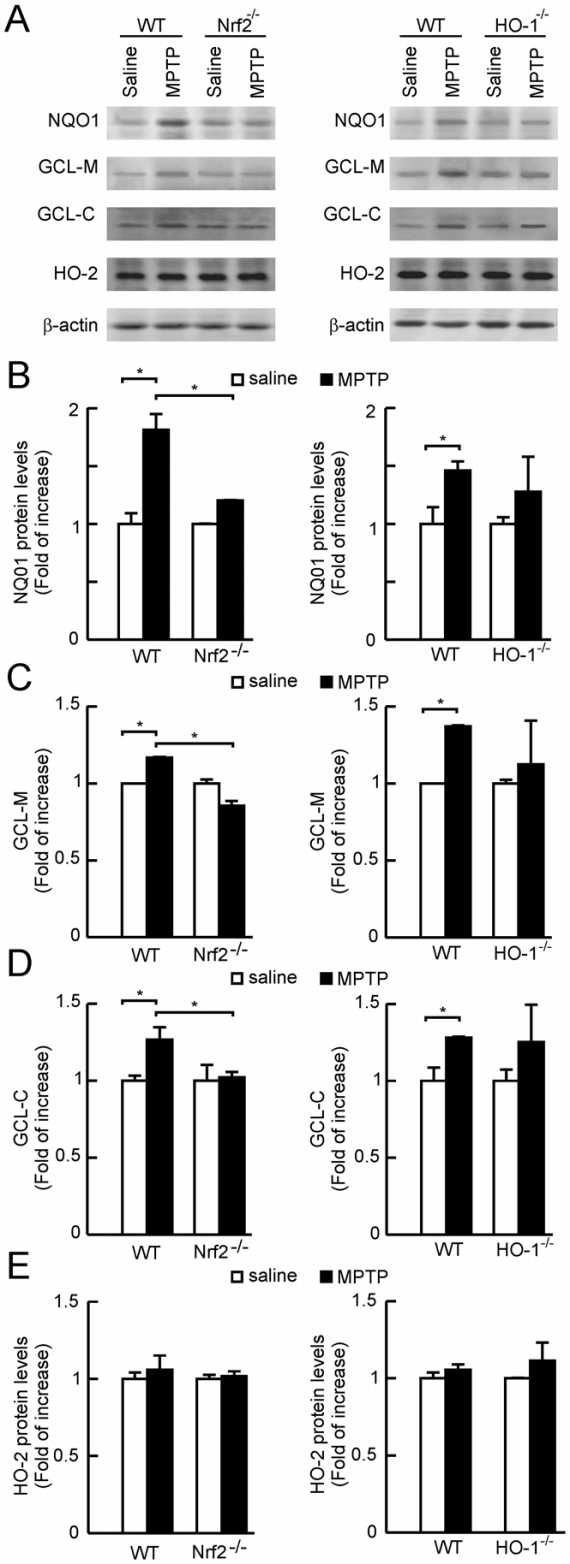
Impairment of the phase II response in Nrf2^−/−^ but not in HO-1^−/−^ mice. *A*, immunoblots showing STR protein levels of the phase II proteins NQO1, GCL-M and GCL-C and the constitutively expressed enzyme HO-2, in Nrf2^−/−^ mice, HO-1^−/−^ mice and their wild type controls under saline or MPTP treatment. *B*, *C*, *D* and *E*, densitometric quantifications (arbitrary densitometry units) from representative immunoblots of *A* after normalization by β-actin densitometry units obtained from the same immunoblots.Results are representative of 5–10 animals per group. Values represent mean ± SD. Two-way ANOVA followed by Bonferroni's test was applied to determine the significance of biochemical differences among groups. Asterisks denote significant differences between treatments with *P*<0.05.

Protein levels of phase II enzymes NQO1, and the two subunits of γ-glutamylcysteine synthetase, GCL-M and GCL-C, were analyzed in STR of Nrf2^−/−^, HO-1^−/−^ and wild type mice (Fig. 7). Protein levels were normalized by β-actin. In wild type and HO-1^−/−^ mice, MPTP led to small increases of less than 1.5 fold in NQO1, GCL-M and GCL-C, which were in the limit of statistical significance. By contrast, in Nrf2^−/−^ mice, none of these three phase II proteins were altered by the MPTP treatment. Moreover, striatal protein levels of HO-2, which is not a phase II enzyme, were not altered by either the genotypic alterations or the MPTP treatment. These results suggest that Nrf2 deficiency produces a stronger impairment in the phase II defense than HO-1 deficiency.

### Iron levels in wild type, Nrf2^−/−^ and HO-1^−/−^ mice submitted to MPTP

**Figure 8:**
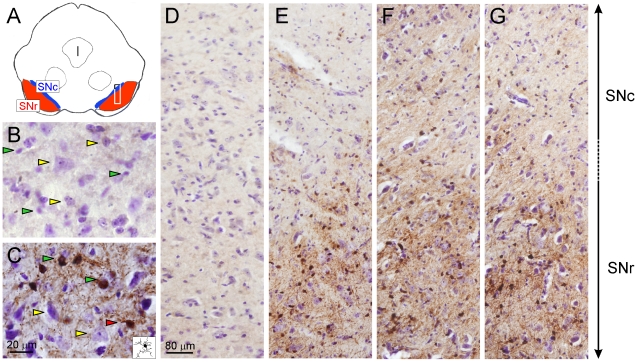
MPTP induces a similar deposition of ferric iron in Nrf2^−/−^ and HO-1^−/−^ mice. Iron precipitates were detected by DAB-enhanced Perls reaction followed by Nissl counterstaining. *A,* simplified scheme of a coronal section of midbrain showing the location of mouse dorsal and ventral substantia nigra on the left (SNc and SNr, respectively). The rectangle on the right side indicates the region shown in panels *D, E, F and G*). *B* and *C*, high magnification pictures of the boundary between SNc and SNr from wild type mice submitted to saline or MPTP treatments, respectively. Yellow arrowheads point some large Perls-negative cells with pale nuclei and dark nucleoli typical of dopaminergic neurons. Green arrowheads point small Perls-positive cells with picnotic nuclei typical of microglia. The red arrowhead points a Perls-positive microglial cell that has been drawn in the inset to show microgrial extensions. The large panels show representative fields of both SNc and SNr (location indicated in the rectangle of *A* panel), from saline-treated wild type mice (*D*), MPTP-treated wild type mice (*E*), MPTP-treated Nrf2^−/−^ mice (*F*), and MPTP-treated HO-1^−/−^ mice (*G*).

Iron levels were measured by 3′-3′-diaminobenzidine-enhanced Perls staining in 40 µm-thick coronal sections from VMB counterstained with Nissl staining. Under these condition ferric ion deposits are stained in brown. As shown in Fig. 8B and 8C, MPTP induced a substantial increase in Perls staining. Most of iron staining was localized in very small, less than 10 µm diameter cell bodies with picnotic nuclei that correspond to microglia and microglial extensions (see inset in Fig. 8C). Very little Perls staining was observed on big cells with big pale nuclei and dark blue nucleoli typical of dopaminergic neurons. Hence, most of iron staining was located in microglia. Then, in the lower magnification panels (Fig. 8D, 8E, 8 F and G) we compared Perls staining along the substantia nigra of saline and MPTP treated mice. There were not significant differences among saline-treated Nrf2^−/−^, HO-1^−/−^ and their respective wild type controls (data not shown). Most of the Perls staining localized to the ventral SN equivalent to pars reticulate. Interestingly, when we compared the Perls staining among MPTP-treated wild type, Nrf2^−/−^ and HO-1^−/−^ mice we found that both Nrf2^−/−^ and HO-1^−/−^ mice exhibited higher Perls staining. The difference with wild type mice was more obvious in the dorsal SN (equivalent to pars compacta). While these results are in agreement with the widely documented iron deregulation in Parkinson's disease, they also indicate that iron deposition is not due to HO-1 activity which is low in Nrf2^−/−^ mice or non-existent in HO-1^−/−^ mice.

## Discussion

In recent years, we and others have hypothesised that the adaptive phase II response, which involves increased expression of destoxification and antioxidant genes, might be a feasible therapeutic target to reduce or stop neuronal death and inflammation in the basal ganglia of PD patients. We have focused on the role of Nrf2, master regulator of the whole phase II response, and HO-1 a polemic phase II enzyme that has been speculated to be both neuroprotective and neurotoxic in PD pathology.

Our first observation was that the MPTP model used here reproduces a well described anatomopathological feature of PD, which is the increased expression of HO-1 in the SN. In our case, MPTP led to increased levels of HO-1 both in mouse STR and VMB that was ascertained to both dopaminergic and non-dopaminergic neurons. We attribute HO-1 up-regulation to an attempt to keep up with redox homeostasis under conditions of oxidative stress. In fact other phase II enzymes such as NQO1 are also up-regulated in this pathological setting [37] [42]. However, since the capacity to induce the phase II response declines with age [13]–[14], which is the main risk factor for PD, it is anticipated that such response needs to be reinforced pharmacologically [17].

Once we established that MPTP elicited a phase II response in the basal ganglia, we analyzed the role of Nrf2 and HO-1 in the pathology. Nrf2^−/−^ mice exhibited increased dopaminergic nigrostriatal degeneration, compared to wild type littermates, as determined by stereological count of nigral dopaminergic neurons, immunohistochemical TH-staining in STR and SN and by HPLC determination of striatal DA and DOPAC. Moreover, basal and MPTP-induced astrogliosis and microgliosis were exacerbated in Nrf2^−/−^ mice. These results are in agreement with previous findings [24]–[25] [43] and further support the concept that phase II induction may provide neuroprotection by acting directly against oxidative stress or by attenuating the chronic inflammation that characterizes this disease.

In contrast to the results obtained with Nrf2^−/−^ mice, the severity of dopaminergic degeneration and neuroinflammation in HO-1^−/−^ mice was comparable to the one observed in their wild type littermates. HO-2 protein levels were not up-regulated in HO-1^−/−^ mice, therefore excluding this trivial compensatory mechanism. Therefore, these observations suggest that Nrf2 deficiency, that is capable of activating expression of the main players of the antioxidant response, is more important for maintenance of cytoprotection against MPTP than the deficiency of one of the components of this response represented by HO-1. Among other Nrf2-regulated genes that might be relevant in this scenario we analyzed the induction of NQO1, and the catalytic and regulatory subunits of γ-glutamylcysteine synthetase (GCL-C and GCL-M respectively) which is the rate limiting enzyme in glutathione synthesis. NQO1 may be relevant to dopaminergic cells due its relevance in reduction of oxidized catechol ring of dopamine. Maintenance of glutathione levels is crucial for nigral dopaminergic neurons. In fact, by using a transgenic mouse in which GCL-C down-regulation is induced in catecholaminergic neurons, including those of the SN, it has demonstrated that reduction in GSH levels in adult dopaminergic midbrain neurons results in nigrostriatal degeneration [44].

Another important objective of our study was to determine if HO-1 induction is neurotoxic or to neuroprotective. Our hypothesis was that if HO-1 is an effector of MPTP-induced dopaminergic cell death, HO-1^−/−^ mice should be more resistant to MPTP-toxicity. On the other hand, if HO-1 is neuroprotective, then HO-1^−/−^ mice should exhibit a stronger dopaminergic lesion. Perhaps surprisingly, we have found that HO-1 deficiency does not modify MPTP toxicity in either way. This finding led us to analyze iron levels, since numerous studies have shown that there is a progressive accumulation of free iron in PD patients [38] [45]–[46]. We found that MPTP treatment increases iron deposition in VMB, as it was described previously [47]. Also, it is well reported that MPTP increases the expression of several iron metabolism regulatory proteins, inflammatory and pro-apoptotic genes while antioxidant defence genes are down-regulated [48]–[49]. The fact that iron is accumulated to a similar extent in HO-1^−/−^ mice and wild type littermates brings up the question if the widely reported iron increase in SN of PD patients is indeed the result of HO-1 activity. However, the evidence supports the idea of a state of oxidative stress in the SN, resulting in a selective accumulation of iron in this particular area [50]. On the other hand, while heme degradation will produce free iron, it will be necessary to examine the contribution of HO-2, which is very abundant in the brain, compared to HO-1. In fact, new evidences suggest that part of HO-1 cytoprotective activity may be independent of its catalytic activity, and related to translocation to the nucleus [51] or mitochondria [52]. Even if free iron is a result of heme oxygenase activity, its accumulation may reflect deficiencies in clearance mechanisms such as divalent metal transporter-1 or ferritin mediated cellular export, transferrin sequestration, iron supply by lactoferrin, etc [53], [54]. In any case, our results largely reduce the relevance of HO-1-mediated iron release in PD pathology.

An important corollary of our study is that pharmacological targeting to Nrf2 in the basal ganglia may be a feasible therapeutic strategy excluding the concern about HO-1-mediated toxicity.
